# In-silico analysis of interacting pathways through KIM-1 protein interaction in diabetic nephropathy

**DOI:** 10.1186/s12882-022-02876-7

**Published:** 2022-07-18

**Authors:** F. Abid, Z. Rubab, S. Fatima, A. Qureshi, A. Azhar, A. Jafri

**Affiliations:** 1 Department Physiology, Jinnah Sindh Medical University, Karachi, Pakistan; 2 Ziauddin Medical College-Ziauddin University, Karachi, Pakistan; 3 Department of Biological and Biomedical Sciences, Aga Khan University, Karachi, Pakistan

**Keywords:** *KIM1*, *HAVCR1*, Diabetic nephropathy, Differentially expressed gene, Hub gene, And immune pathways

## Abstract

**Background:**

Human Kidney Injury Molecule-1, also known as HAVCR-1 (Hepatitis A virus cellular receptor 1), belongs to the cell-surface protein of immunoglobulin superfamily involved in the phagocytosis by acting as scavenger receptor epithelial cells. The study focused on pinpointing the mechanisms and genes that interact with KIM-1.

**Methods:**

This in-silico study was done from March 2019 to December 2019. The Enrichment and protein-protein interaction (PPI) network carefully choose proteins. In addition, the diagramed gene data sets were accomplished using FunRich version 3.1.3. It was done to unveil the proteins that may affect the regulation of HAVCR1 or may be regulated by this protein. These genes were then further considered in pathway analysis to discover the dysregulated pathways in diabetic nephropathy. The long list of differentially expressed genes is meaningless without pathway analysis.

**Results:**

Critical pathways that are dysregulated in diabetic nephropathy patients have been identified. These include Immune System (Total = 237, *P* < 0.05), Innate Immune System (Total = 140, P < 0.05), Cytokine Signaling Immune system (Total = 116, P < 0.05), Adaptive Immune System (Total = 85) and Neutrophil degranulation (Total = 78).

**Conclusion:**

The top 5 genes that are interacting directly with *HIVCR1* include *CASP3, CCL2, SPP1, B2M, and TIMP1* with degrees 161, 144, 108, 107, and 105 respectively for Immune system pathways (Innate Immune System, Cytokine Signaling Immune system, Adaptive Immune System and Neutrophil degranulation).

## Introduction

Diabetic nephropathy (DN) is well identified because of its potential to lead to end-stage renal disease (ESRD) that commonly occurs due to complications related to diabetes mellitus [1]. Generally, tubule-interstitial injury commonly occurs in all chronic kidney diseases in addition to diabetes. In DN, complex changes in early adaptive renal structure ensue, such as tubular and glomerular hypertrophy that have been observed along with the accumulation of components of glomerular mesangium extracellular matrix and tubulo-interstitium [2].

In patients, early detection of the tubular lesion can help to manage DN through glycemic control. Further, glomerular involvement is followed mainly by tubular involvement as various tubular enzymes and proteins are noticeable even before serum creatinine levels increase and the appearance of microalbuminuria [3]. Moreover, serum urea nitrogen, creatinine, and urinary protein levels can reveal changes in infiltration capacity, but they do not act as actual markers of ‘injury’ [4].

A HAVCR1 protein with receptors on the surface of the immunoglobulin superfamily was found to be involved in phagocytosis, acting as scavenger receptor epithelial cells [5]. There is a six-cysteine immunoglobulin-like domain in its extracellular portion, consisting of mucin-like O- glycosylated proteins with a threonine/serine and proline-rich and two N- glycosylation sites [6]. KIM comprises one transmembrane, an extracellular, and an intracellular domain [7]. KIM-1 can oxidize lipoproteins and is also involved in identifying apoptotic cell surface-specific epitopes, phosphatidylserine [8]. KIM-1 intercedes dead and necrotic debris engulfment in the injured epithelial tubules [9]. It facilitates phagocytosis through cell surface binding and activating internalization, but that epithelial cell transforms into semi-professional phagocytes. It supports evading exfoliated cells’ unwanted attachment to fibronectin and diminishes tubular obstruction and cast development [10]. These characteristics might make KIM-1 kidney injury as an ideal biomarker. The project’s primary focus is identifying the genes/pathways that interact directly or indirectly with KIM-1.

The study used a network-based pathway enrichment methodology for detecting KIM-1 associated biological processes. This method was performed based on a known pathway network and biological network analysis. Dysregulated pathways were also identified based on pathway network and target network analysis to elaborate the importance of KIM-1 in diabetic nephropathy.

## Methodology

This is an in-silico study, completed in between March, 2019 to December, 2019. Enrichment and protein-protein interaction (PPI) network exploration of the notorious proteins, alongside mapped gene datasets, was executed using FunRich version 3.1.3.

This study was followed by a study done by one of the authors on KIM-1 [11]. Kidney Injury Molecule-1 (KIM-1) peptide is specific to tubular hurt in the blood. It assessed kidney injury molecule-1 level in people with diabetes with a history of kidney disease and no history and evaluated screening accuracy of KIM-1. This prospective study design comprised *n* = 85 subjects as duplicate samples from the diabetic unit of Jinnah Post-graduate Medical Center (JPMC) and Nephrology department in cooperation with Aga Khan University Hospital (AKUH) from November, 2016 to September, 2017. The ethical evaluation board of Basic Medical Sciences Institute, JPMC Karachi (Ref NO.F.2–81-IRB/2017/GENL/419/JPMC), Pakistan, granted the study’s ethical approval.

### Gene expression data

This study obtained the E-GEOD-30529 gene expression profile 9 [12] from the Array Express database [13], based on the Affymetrix GeneChip Human Genome U133A 2.0. The datasets of E-GEOD-30529 consists of 22 samples comprising 10 Diabetic Kidney Disease patients, whereas the remaining 12 samples are associated with standard conditions.

### Data pre-processing and detection of DEG

Series matrix of the dataset with accession to E-GEOD is downloaded from geo and is processed to convert to an .xlsx file as SAM accepts the input file in this format. The DEG was identified through the SAM (Significant Analysis of microarray) method [14]. The genes with a delta value of 1.27 and fold change 1.35 are considered as differentially expressed. Two thousand seven hundred and twenty-nine differentially expressed genes are identified, from which the top 2000 genes are considered for further analysis.

### Construction of the target network

DEG (Differentially expressed genes) pair’s possible functional relationships were investigated through the search tool STRING database for retrieving the interacting genes/proteins [15]. This network was constructed by giving the DEGs as input to STRING for the retrieval of interactions among these genes. The genes and their interactions were extracted and used to run a network analysis.

### Network analysis of topological network and extraction of the target network

Cytoscape 3.8.2, a free software tool, was used for analyzing, modeling, and visualizing the interactions network at molecular and genetic levels [16]. In this article, Cytoscape 3.8.2 was used to characterize the biological importance of genes over the index of topological centrality. Each gene degree quantified the local topology by adding the adjacent genes’ numbers. It gives a single node count through the number of interactions. The high degree node suggests a central node in the network of interactions [17]. If a degree associated with a particular gene was ranked in the top 5 among the genes in the target network, the gene was considered the hub gene.

### Construction of pathway network

STRING is an online application devoted to protein-protein interactions. It comprises physical (direct) and functional (indirect) interactions. It contains publicly available databases, experimental sources, co-expression, and genomic perspectives. In this database, 24,584,628 proteins from 5090 organisms were included at this time [15]. The REACTOME database was used to obtain the biological pathways data [18]. In this database, a total of 2441 pathways were included. Each pathway was extracted based on the gene pairs from the total gene pair according to the pathway genes.

## Results

### Identification of differentially expressed genes

Statistically significant genes in diabetic nephropathy are discovered using the SAM technique. About 2571 genes are discovered as differentially expressed 1765 genes and are up-regulated in the diseased as compared to control, whereas 805 genes are down-regulated in the diseased group. HAVCR1 is positively differentially expressed with a delta value of 2.544952, and a fold changes the value of 1.353374. Top 2000 genes are considered for further analysis.

### Construction of topological network

Two thousand genes are given to STRING to create an interaction network. The STRING network consisting of 21,975 edges and 1634 nodes is retrieved with the confidence of 0.400.

### Network analysis and selection of target network

The interaction file is later imported into Cytoscape network analysis. Network analysis is performed to compute the topological parameters of the networks. After performing the network analysis, only the genes interacting directly or indirectly with the gene of interest, i.e. HAVCR1, are selected. The selection is completed in two steps. The nodes and edges directly connected to HAVCR1 are selected at the first point. The nodes and edges connected to the first neighbors are selected in the next step. We cannot analyze the complete network obtained from STRING, only the nodes connected directly or indirectly to our gene of interest are extracted.

**Fig. 1:**
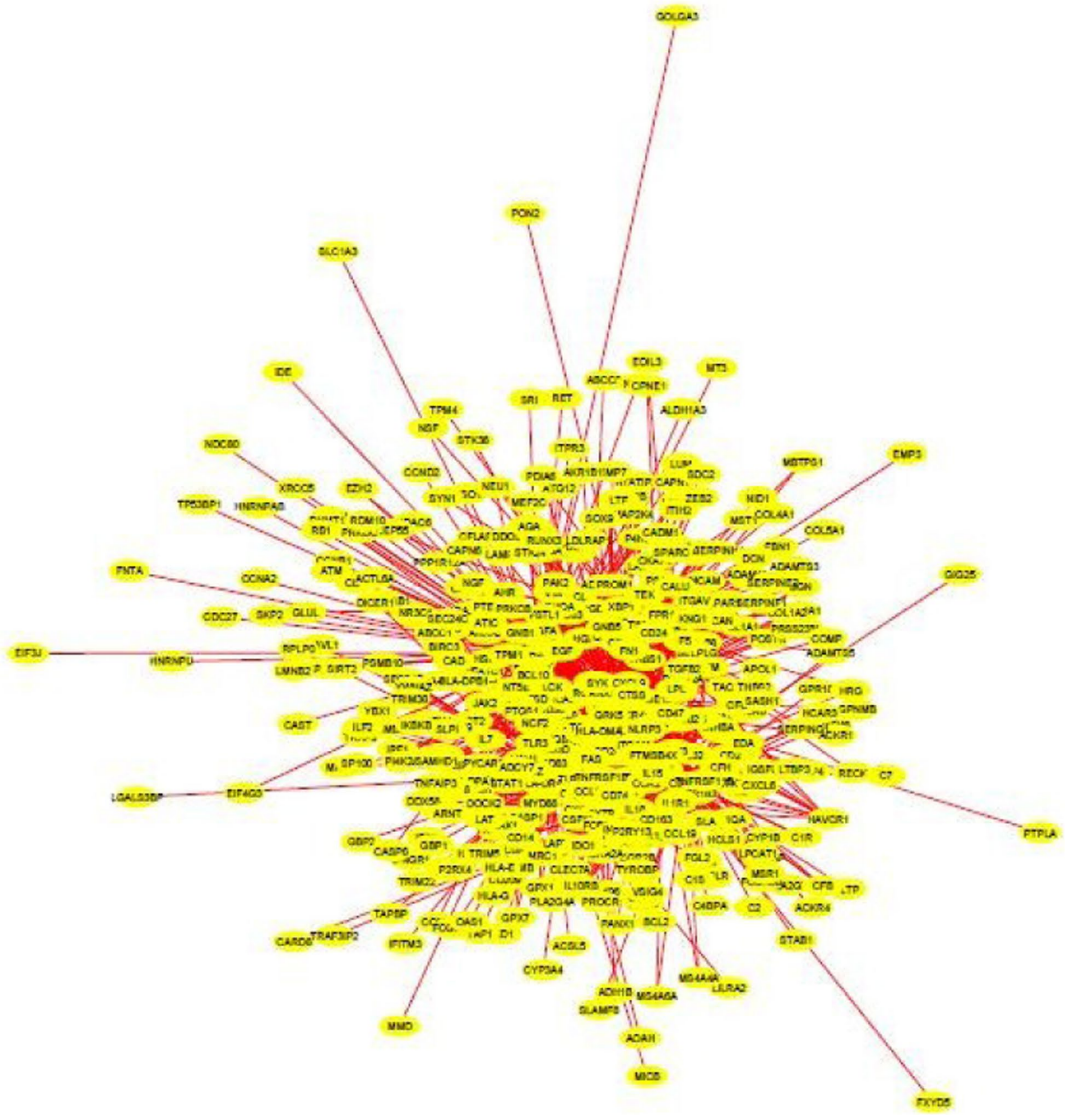
Target network comprising of HAVCR1 and its neighbors

The top 5 genes interacting directly with HAVCR1 include CASP3, CCL2, SPP1, B2M, and TIMP1 with degrees 161, 144, 108, 107, and 105, respectively. The top ten genes and their genes are displayed in Table 1. About 450 genes are identified to interact directly or indirectly with the protein of interest. The topological network of HAVCR1 and its neighboring genes is shown in Fig. 1. The number of edges in the target network is 941, and the number of nodes is 941.

**Table 1:** HAVCR1 and its neighboring genes

Gene	Degrees
*HAVCR1*	10
*CCL2*	144
*LCN2*	40
*CLU*	66
*TIMP1*	105
*IL18*	90
*C3AR1*	103
*B2M*	107
*CASP3*	161
*SPP1*	108
*HPGDS*	45

### Pathway analysis

Approximately 85 pathways enriched in differentially expressed genes with *P*-Value < 0.05 were found using this pathway analysis. Out of these, 26 pathways have neighboring genes. Later, the number of neighboring genes is calculated by taking the intersection of genes in the pathway and the neighboring genes. The top 5 pathways include Immune System, Innate Immune System, Cytokine Signaling in Immune System, Adaptive Immune System, and Neutrophil Degranulation, with a neighbouring gene count of 237, 140, 116, 85, and 78 respectively. The top 8 pathways and the number of neighboring genes are shown in Table 2.

**Table 2:** Major deregulated pathways and genes involved

Pathways	Number of hub genes involved	Genes
Immune System	237	*TIMP1, HGF, CTSD, LYZ, VWF, EGF, SERPING1, CLU, C1R, HSPA5, CFH, CFB, C1S, B2M, QSOX1, RHOA, CFD, VEGFA, FN1, BCL2, CD44, ITPR3, FCGR2A, VCAM1, PAK2, LCK, ACTB, SYK, HCK, LYN, ITGB1, VAV1, CYFIP1, ATIC, CCL2, CCR2, CXCL8, CCL19, CCL5, CXCL1, ANXA1, CCL20, FCGR2B, CASP8, LAMP2, LPCAT1, CASP3, VCL, COL1A1, IRF1, TLR2, ITGAV, SAMHD1, ICAM1, TLR3, COL1A2, PDIA3, CD28, P4HB, CASP9, HMGB1, ANXA2, TLR7, CD63, KRT8, STAT1, CYBB, ALOX5, ITGAM, PTEN, VIM, SQSTM1, PTPRC, PECAM1, MX1, ALOX5,ITGAM,PTEN,VIM, SQSTM1, PTPRC, PECAM1, MX1,PPIA, HSP90AB1, RPLP0, MYC, YWHAZ, ITGB3, IRF2, ILF2, ITGB2,CAPN1,CD59, HLA- DQB2,,C3, PYCARD, AGA,CD3D, IL7R, PANX1,CDA, ADAM10, STK10,CMTM6, MICB, SNAP25, MYD88,ADAM17, FCER1G, LILRB2, CKAP4, C3AR1,CD36,RAP2B, CD53, CD47, TNFRSF1B, PRKCB,SELL, COL3A1, CTSK, IL18, BST2, HLA-F,IRF8, DDX58,HLA-G, HLA-E, HLA-A,HLA-C,HLA-B,GBP2, IFITM3, PSMB8, OAS1, IRF9, GNB5, PSAP, FPR1, CD14, EDA, RAB27A, NLRP3, XRCC5, CLEC7A, JAK1, TYROBP, CD86, NCF2, LCP2, C1QB, CTSS, LY86, CSF2RB, CSTB, MID1, CD209, SLPI, GZMB, TNFSF10,EPO,BIRC3, IL7,CSF1R,CD1D, ATG12,CASP4, IL15, CASP1, JAK2, CFLAR,APAF1, FGL2, LY96,CD74, SEC24D, SEC24C, SIGLEC1, BCL10, CARD8, TRIM5, TRIM22,DDOST, SKP2, CDC27, IL10RA, DOCK2, PRKDC, GAB2, MAP2K4, QPCT, TXNIP,RET,IKBKB,NFATC1, LTF,TNFAIP6, LILRA2, SP100, TAP1, TRIM38, PSMB10, CD247, TLR1, CD1C, FCGR1B, GBP1, LCN2, PSMB9,NEU1, LAT, TAPBP, TNFAIP3,TRAF3,CYLD, C7,IL11, TNFRSF11B, IFI16, GLIPR1, CPNE1,C1QA, C2,C4BPA,FCGR3B, MRC1, CLEC10A,IL10RB, IFNGR1, MEF2C, IL33, EIF4G3*
Innate Immune System	140	*CTSD,LYZ,SERPING1,CLU,C1R,CFH,CFB,C1S,B2M,QSOX1, RHOA, CFD,BCL2, CD44, ITPR3, FCGR2A,PAK2, LCK, ACTB, SYK,HCK,LYN,VAV1, CYFIP1,ATIC, CCR2, CXCL1, FCGR2B, CASP8, LAMP2, LPCAT1, VCL,TLR2, ITGAV, TLR3, CASP9, HMGB1, ANXA2, TLR7, CD63,KRT8, CYBB, ALOX5, ITGAM, PTPRC, PECAM1, PPIA, HSP90AB1, ILF2, ITGB2, CAPN1, CD59, C3,PYCARD, AGA,CD3D,PANX1, CDA,ADAM10, STK10, CMTM6, SNAP25, MYD88, FCER1G, LILRB2, CKAP4, C3AR1, CD36, RAP2B, CD53, CD47, TNFRSF1B,SELL, CTSK, BST2, DDX58, HLA-E, HLA-A, HLA-C, HLA-B,PSMB8, PSAP, FPR1, CD14, RAB27A, NLRP3, XRCC5, CLEC7A,TYROBP, NCF2, LCP2, C1QB, CTSS, LY86, CSTB, CD209,SLPI, GZMB, EPO,BIRC3, ATG12, CASP4, CASP1, CFLAR,APAF1, FGL2, LY96, BCL10, CARD8, DDOST, DOCK2, PRKDC, GAB2, MAP2K4, QPCT, TXNIP, IKBKB, NFATC1, LTF,TNFAIP6, PSMB10, CD247, TLR1, LCN2, PSMB9, NEU1, LAT,TNFAIP3,TRAF3, CYLD, C7,IFI16, GLIPR1, CPNE1, C1QA, C2, C4BPA, FCGR3B, CLEC10A,MEF2C*
Cytokine Signaling in Immune system	116	*TIMP1,HGF,VWF,EGF,B2M,VEGFA,FN1, BCL2, CD44, VCAM1,PAK2, LCK,ACTB, SYK, HCK,LYN, ITGB1,VAV1, CCL2, CCR2, CXCL8, CCL19,CCL5, CXCL1, ANXA1,CCL20,CASP3,VCL, IRF1, SAMHD1, ICAM1,COL1A2, P4HB, HMGB1,ANXA2, STAT1, ALOX5,ITGAM,VIM,SQSTM1, MX1,PPIA, RPLP0, MYC,YWHAZ,ITGB3, IRF2, ITGB2,HLA-DQB2, HLA-DPA1,HLA-DPB1, HLA-DQB1, HLA-DRA,IL7R, SNAP25, MYD88, ADAM17, CD36, TNFRSF1B,IL18, BST2, HLA-F,IRF8,DDX58, GBP2,IFITM3, PSMB8,OAS1, IRF9, FPR1, EDA, JAK1,CD86, CSF2RB, MID1, GZMB, TNFSF10, BIRC3,IL7, CSF1R,IL15, CASP1,JAK2, TRIM5,TRIM22, IL10RA,GAB2, MAP2K4, RET,IKBKB, SP100,TRIM38, PSMB10, FCGR1B, GBP1, LCN2, PSMB9, LAT,TRAF3,IL11, TNFRSF11B, IL10RB, IFNGR1, MEF2C,IL33, EIF4G3, IL1R1,IL32, FNTA, TEK,INPP5D*
Adaptive Immune System	85	*CTSD, HSPA5, B2M,ITPR3, VCAM1, PAK2, LCK,SYK, LYN,ITGB1, VAV1, FCGR2B, COL1A1, TLR2, ITGAV, ICAM1, COL1A2, PDIA3, CD28, CYBB, PTEN, PTPRC, PPIA, YWHAZ,ITGB2, HLA-DQB2,HLA-DPA1,HLA-DPB1,HLA-DMB, HLA-DMA,HLA-DQB1,HLA-DRA,C3,CD3D, MICB, SNAP25, MYD88, LILRB2, CD36, PRKCB,SELL, COL3A1, CTSK, HLA-F,, PSMB8, GNB5, FPR1, CD14, TYROBP, CD86, NCF2,LCP2, CTSS, CD209,TNFSF10,CD1D, LY96, CD74, SEC24D, SEC24C, SIGLEC1,BCL10, SKP2, CDC27, IKBKB, NFATC1, LILRA2,TAP1, PSMB10, CD247, TLR1, CD1C, FCGR1B, PSMB9, LAT, TAPBP, MRC1, IFNGR1, KLRB1, INPP5D*
Neutrophil degranulation	78	*CTSD, LYZ,C1R,B2M,QSOX1, RHOA, CFD,CD44, FCGR2A, CYFIP1, ATIC, CXCL1, LAMP2, LPCAT1, VCL,TLR2, ITGAV, HMGB1, ANXA2, CD63, KRT8, CYBB, ALOX5, ITGAM,PTPRC, PECAM1, PPIA, HSP90AB1,ILF2, ITGB2, CAPN1, CD59,C3,PYCARD, AGA,CDA,ADAM10, STK10, CMTM6, SNAP25,FCER1G, LILRB2, CKAP4, C3AR1, CD36, RAP2B, CD53, CD47,TNFRSF1B, SELL, BST2,, PSAP, FPR1,CD14, RAB27A, XRCC5, TYROBP, CTSS, CSTB, SLPI, GZMB,EPO, APAF1, FGL2, DDOST, DOCK2, QPCT, LTF,TNFAIP6, LCN2, NEU1, GLIPR1, CPNE1, C4BPA, FCGR3B*
Signaling by Interleukins	69	*TIMP1, HGF,VEGFA, FN1,BCL2, VCAM1, PAK2, LCK, SYK,HCK,LYN,ITGB1, VAV1, CCL2, CCR2, CXCL8,CCL19, CCL5, CXCL1, ANXA1, CCL20, CASP3, ICAM1, COL1A2, P4HB, HMGB1, ANXA2, STAT1, ALOX5, ITGAM, VIM, SQSTM1, PPIA, RPLP0, MYC,YWHAZ, ITGB2, IL7R, SNAP25, MYD88,CD36, TNFRSF1B,IL18, PSMB8, FPR1, JAK1, CD86, CSF2RB, GZMB, IL7,CSF1R, IL15, CASP1, JAK2, IL10RA, GAB2,MAP2K4, IKBKB, PSMB10, LCN2, PSMB9,IL11, IL10RB, IFNGR1, MEF2C, IL33, IL1R1, IL32, INPP5D*
Extracellular matrix organization	60	*A2M,TIMP1, SPARC, CTSD, MMP7, THBS1, VWF,ACTN1,TGFB2, FN1,CD44, VCAM1, ITGB1, COMP, CASP3, ITGB8, COL1A1, ITGAV, ICAM1, ITGA2, COL1A2, P4HB, SPP1, ITGAM,PECAM1, ITGB3, ADAM12, ITGB2, CAPN1, ADAM10, LAMB1, LAMC1,ADAMTS5,ADAMTS3,ADAM17, TNC,SDC2, VCAN, MATN3, CD47, FBN1, COL5A1, MMD, DCN,FMOD, BGN,COL3A1, LUM,CTSK, BMP7, CTSS, GZMB, CAST, SERPINH1,CAPN6, LAMC2,IKBKB, NID1, COL4A1, LTBP3,*
Platelet activation, signaling and aggregation	52	*A2M, TIMP1, HGF, SPARC, HRG, SRGN, THBS1, VWF, KNG1, EGF, SERPING1, CLU, HSPA5, TOR4A, QSOX1, RHOA, TMSB4X, CFD, ACTN1, VEGFA, F5, TGFB2, FN1, ITPR3, LCK, SYK, LYN, VAV1, LAMP2, VCL, COL1A1, ANXA5, COL1A2, CD63, PECAM1, PPIA, YWHAZ, ITGB3, PLEK, FCER1G, CALU, CD36, PRKCB, GNB3, GNB5, ADRA2A, PSAP, GNB1, PLA2G4A, LCP2, LAT, LGALS3BP*

## Discussion

Pathogenesis of DN is complex, and pathological renal lesions are diverse. Diagnosis and progression of diabetic nephropathy (DN) have long been monitored through glomerular filtration rate (GFR); the kidney’s best functional marker. But it was time consuming and was typically calculated from equations that rested on serum creatinine and cystatin C. Microalbuminuria and proteinuria have been another non-invasive biomarker. Still, their limitations include that patients having advanced kidney disease and microalbuminuria detected in them do not respond to therapy, as it would be at an earlier stage. Further, the ultra-filtered proteins cause advanced tubulo-interstitial damage to the kidney [19, 20]. Diabetic nephropathy embraces events causing the destruction of filtration membrane and capillaries and causing the kidney function to be impaired [21].

Other disparities include mesangial cell expansion, Kimmelstiel–Wilson nodules, glomerulosclerosis, and interstitial tubular fibrosis [22]. Reasons behind this are due to both hereditary and ecological factors linked with diabetes, with the most noteworthy being high blood glucose and high blood pressure. Primary functional irregularity is intraglomerular hypertension, hyperfiltration and is supplemented by the onset of microalbuminuria [23]. The cellular components of the kidney react to hyperglycemia in several ways, namely hemodynamic, metabolic, and inflammatory [24, 25]. There are three pathways involved, including the hemodynamic pathway which is based on efferent arteriolar constriction culminating in the raised intraglomerular pressure because of initiation of the renin-angiotensin-aldosterone-mechanism vessels, as well as hyperfiltration [25]. The renin-angiotensin-aldosterone-system is activated by hyperglycemia in diabetic patients. Angiotensin II is responsible for initiating inflammation, renal cell growth, and self-digestion/degradation of podocytes [23]. Glomerular perfusion and glomerular filtration rate (GFR), typically kept in place by an autoregulatory mechanism, are also deranged and perturbed by the prolonged elevated glucose levels. This process, although initially causing increases in GFR, later on leads to the thickening of endothelial mesangial cells [21].

Another main pathway is the metabolic pathway. It contributes to the pathogenesis of diabetic nephropathy by excessive glucose degradation among cells – a process called ‘glycolysis’. This enhances the polyol pathway in which there is an excessive formation and storing of sorbitol in cells. Sorbitol lowers nitric oxide, thus leading to intracellular stress and apoptosis. Next to this impact is the formation of fructose from sorbitol which is highly nephrotoxic. Fructose directly causes protein loss from kidneys and lowers the glomerular filtration rate and abundance of superoxide ions and other inflammatory cytokines [25]. Second to this is hexosamine pathway stems, which is a by-product of glycolysis, tumor necrosis factor-a, and transforming growth factor-B1. All this ends in hypertrophy of nephrocytes [25]. Advanced glycation end-products, which enhance vascular complications of diabetes mellitus, are shaped through the advanced glycation of proteins in the renal cells. These products, when accumulated around the tubulo-interstitial cells and glomeruli in the kidneys, further lead to hardening of the glomerular basement membrane and fibrosis of these structures [23].

Advanced glycation end-products also are capable of attaching to pro-inflammatory receptors; altering the actions of renal cells also gives rise to cytokines and reactive oxygen species (ROS) which are nephrotoxic [25]. The last step of glucose degradation is the stimulation of protein kinase C (PKC) which is formed from glyceraldehyde-3-phosphate. This production of PKC directly leads to the initiation of two vasodilator substances, prostaglandin E2 and nitric oxide (NO) [25]. This leads to an increase in glomerular filtration, as mentioned earlier.

It has also been documented that activated protein kinase C leads to glomerular basement membrane stiffening and the buildup of the extracellular matrix, which both affects the permeability of capillaries and, in turn, large amounts of albumin enter the vessels [23]. Lastly, the most critical and contributing pathway related and justified by our analysis is the inflammatory immune pathway. The chronicity of diabetes mellitus leads to inflammatory signals that affect the renal cells and cause modification and remodeling and trigger the innate and adaptive immune response [26, 27]. Together, releasing inflammatory cytokines engender the enhanced glomerular basement membrane thickening, fibrosis, and increased permeability of capillaries [28]. Kidney damage resulting from high blood glucose levels in diabetic patients is known as diabetic nephropathy. The expression levels of KIM-1 and AER (albumin excretion rate) can act as a diabetic nephropathy prognostic marker [29]. Many studies show that KIM-1 can act as a diabetic nephropathy prognostic marker. The purpose of carrying this research is the identification of deregulated pathways through KIM-1 interaction.

Many diabetic patients are observed to suffer from kidney-related diseases and inflammation. In several experimental studies, CCL2 is proposed as a potential healing target and biomarker in renal tissue-impaired patients having diabetes [30]. LCN2 is predicted as a significant early biomarker to diagnose DN at early and developing stages [31]. CLU is experimentally observed to be overexpressed in DN, thus predicted as a therapeutic target and potential biomarker of DN. TIMP1 is experiential to show an essential part in the advancement of DN due to its under-expression [32]. IL18 (interleukin-18) has a significant role as an inflammation mediator in the development and progression of nephropathy. IL18 is reported to be overexpressed in renal tissues in diabetic nephropathy [33]. C3AR1 (Complement C3a Receptor 1) is involved in inflammatory responses and is overexpressed in diabetic patients. It is experimentally demonstrated in a study that knockout of C3AR1 in mice slows down the progression of DN [34]. B2M (beta-2-microglobulin) is overexpressed in kidney-related diseases. It is reported as an early biomarker in DN development [35]. CASP3 is proposed as a significant therapeutic target in DN. The inhibition of CASP3 in mice resulted in exacerbating symptoms of DN. Consequently, it could be treated as a potential therapeutic target [34]. SPP1 is experimentally reported as a differentially excreted protein in DN patients [35].

## Conclusion

In this study, crucial mechanisms were brought to focus using protein-protein interaction of networks, centered on the top 5 genes interacting directly with HAVCR1, consisting of CASP3, CCL2, SPP1, B2M, and TIMP1. These are essential for immune system pathways (Innate Immune System, Cytokine Signaling Immune system, Adaptive Immune System Pathways, and Neutrophil Degranulation), and were established to be amplified in diabetic nephropathy through protein interaction studies. These signaling pathways and associated proteins serve to be a potential target for novel beneficial agents to decrease the burden of diabetic nephropathy resulting from chronic diabetes mellitus.

## Data Availability

Availability of data and materials. The datasets generated and/or analyzed during the current study is available from the corresponding author on reasonable request.
