# Correction: In-silico analysis of interacting pathways through KIM-1 protein interaction in diabetic nephropathy

**DOI:** 10.1186/s12882-022-02917-1

**Published:** 2022-08-18

**Authors:** F. Abid, Z. Rubab, S. Fatima, A. Qureshi, A. Azhar, A. Jafri

**Affiliations:** 1 Department Physiology, Jinnah Sindh Medical University, Karachi, Pakistan; 2 Ziauddin Medical College-Ziauddin University, Karachi, Pakistan; 3 Department of Biological and Biomedical Sciences, Aga Khan University, Karachi, Pakistan; 4 Biochemistry Department of Jinnah Sindh Medical University, Karachi, Pakistan

**Correction: BMC Nephrol 23, 254 (2022)**

**https://doi.org/10.1186/s12882-022-02876-7**

Following publication of the original article [1], we have been informed that Dr. Jafri has been incorrectly affiliated.

The incorrect affiliation is: Department of Biological and Biomedical Sciences, Aga Khan University, Karachi, Pakistan.

The correct affiliation is: Biochemistry Department of Jinnah Sindh Medical University, Karachi, Pakistan.

The original article has been corrected.
